# Genetic risk and plasma biomarkers of dementia with Lewy bodies in a Chinese population

**DOI:** 10.1002/alz70855_100125

**Published:** 2025-12-23

**Authors:** Xiaoli Hao, Xuewen Xiao, Ling Weng, Jifeng Guo, Junling Wang, Xinxiang Yan, Beisha Tang, Jinchen Li, Bin Jiao, Lu Shen

**Affiliations:** ^1^ Xiangya Hospital, Central South University, Changsha, Hunan, China

## Abstract

**Background:**

Genetic investigations and association with plasma biomarkers of dementia with Lewy bodies (DLB) in East Asian populations are lacking.

**Method:**

This cohort included 151 DLB patients and 2010 controls, all of whom underwent whole genome sequencing. The associations were validated in the ChinaMAP database. Plasma glial fibrillary acidic protein (GFAP), α‐synuclein(αSyn), neurofilament light (NfL), and phosphorylated tau 217 (*p*‐tau217) were detected in a subgroup.

**Result:**

The *APOE* ε4 allele significantly increased DLB risk (*p* = 1.84E‐11), while rare missense variant of *USP13* gene was first found to be associated with DLB risk (*p* = 1.31E‐5). Higher levels of plasma GFAP, αSyn, NfL, and *p*‐tau217 were detected in DLB patients (*p* < 0.001), which combined polygenic risk scores (PRS) achieving an AUC of 0.927 for DLB diagnosis. Besides, significant correlations were found between PRS of DLB and age at onset, the cumulative incidence rate, as well as plasma GFAP level. Plasma NfL level was positively associated with the atrophy of bilateral temporal lobe.

**Conclusion:**

This research is the first genetic investigation of DLB in East Asia. We found that *APOE* ε4 and rare variant of *USP13* gene significantly increased the risk of DLB, highlighting the discriminative ability for DLB using PRS and plasma biomarkers assay.

